# Supplementary material for: Are carbon emissions trading and green financial instruments synergistic? -Comprehensive quantitative research based on content analysis
Source: PLoS One. 2024 Mar 7;19(3):e0298601. doi: 10.1371/journal.pone.0298601 (PMC10919601; doi:10.1371/journal.pone.0298601)
Supplement: S1 Data — (ZIP) [file pone.0298601.s001.zip › Data/地级市Z_F工作报告.zip!/║■▒▒/╩«╤▀╩╨/└┤╫╘╣1⁄2╓┌║┼í╛┬φ┐╦╩2╛▌═°í┐_2004.docx]

各位代表：

　　现在，我代表市人民政府向大会作工作报告，请予审议，并请各位政协委员和列席会议的同志提出意见。

　　一、2003年工作回顾

　　2003年，在市委的正确领导下，全市上下认真贯彻落实党的十六大和十六届三中全会精神，团结拼搏，克难奋进，经受住了突发非典疫情和特大洪水等自然灾害的严峻考验，积极应对汽车市场结构性变化带来的不利影响，国民经济保持平稳增长，各项社会事业全面发展，政治文明和精神文明建设迈出新的步伐。全市实现生产总值242.3亿元，同比增长4.1%，其中第一产业增长4.79%，第二产业下降1.9%，第三产业增长13.9%；实现财政收入23.3亿元，增长11.6%，其中地方一般预算收入8.5亿元，增长6.1%；完成固定资产投资62.7亿元，增长23.6%；直接利用外资3050万美元，增长26.8%；实现社会消费品零售总额93.2亿元，增长11.9%；城镇居民人均可支配收入6217元，净增357元,增长6.1%；农民人均纯收入1808元，净增94元，增长5.5%；城乡居民储蓄存款净增31亿元，增长20.3%，人均储蓄存款净增900元。生产总值未能完成年初计划，国民经济的部分指标增幅高于往年，其中一些指标增幅居全省前列。

　　1、县域经济快速发展。坚持“一主三化”方针，大力发展县域经济。五县一市实现生产总值98.7亿元，增长7.6%，比全市增幅高3.5个百分点；实现财政收入8.2亿元，增长12%；完成固定资产投资27.1亿元,增长47.7%；民营经济增加值67.6亿元, 民营经济占生产总值比重达到68.4%，提高2.3个百分点；县域工业化率达到28.8%,同比提高1.5个百分点。农村经济稳步发展。全市实现农业总产值51.5亿元，增长4.5%；黄姜、茶叶等特色产业基地面积发展到226万亩，特色产业产值占农业总产值比重提高到77%；乡镇企业实现增加值27.2亿元，实现利润5.2亿元，分别增长15%和14.7%；农民打工收入占人均纯收入的39%。城镇化进程步伐加快。全市城镇化率达到34.6%，提高2.1个百分点。基础设施建设进一步加强。农村电网改造基本结束，修筑通乡油路944公里、通村公路318公里；建沼气池1.37万口；建设饮水工程1.53万处，解决了9万人饮水困难。新阶段扶贫开发工作扎实有序推进，283个重点贫困村建设全面展开。

　　2、各项改革不断推进。以产权制度改革为重点的国有集体企业改革稳步推进。全市865家企业完成改制任务，其中当年改制480家，改制面达到90.8%。市公交公司完成合资改制任务，我市率先在全国实现了城市公交整体民营化。全市事业单位全员竞聘、竞争上岗稳步进行。继续深化农村税费改革，推进乡镇综合配套改革。乡镇（街办）内设机构由810个减少到253个，事业单位由2356个减少到782个，农村中小学由3433所调整为2552所。开展农村合作医疗制度改革试点工作，农村医疗卫生条件得到初步改善。深化财政改革，实现了财政收支平衡。金融保险改革平稳推进，金融保险业都有新的发展。

　　3、对外开放成效显著。成功承办第十一届省运会、第六届武当国际旅游节和南水北调中线工程开工邮票首发式活动，多途径招商引资，引进了一批国内外知名公司，促成了开发堵河流域水能资源、高纯度多晶硅、农夫山泉、先锋模具、三立灯具、魔芋系列深加工、香格里拉城市花园等重大项目的签约。全市引进市外境内资金17.4亿元，增长1.45倍。外贸出口完成3548万美元，其中县市区出口2232万美元，增长95.1%。

　　4、项目建设进展顺利。城市建设力度加大。北京路全线贯通，人民路、公园路和南岳路“示范街”建设如期完工，柳林路改造一期工程竣工，东风大道路基工程基本完成，十堰火车站改造完工运营，市体育中心、污水处理厂二期工程建成投入使用，国务院正式批准在十堰设立海关，国家火炬计划汽车关键零部件产业基地落户十堰高新技术开发区，十堰城区顺利通过国家园林城市复查验收，综合大学完成总体方案评审。重点项目建设进度加快。襄十高速公路建成通车，十漫高速公路已经国家正式批准，即将开工建设；武当山机场完成项目设计，前期各项准备工作基本就绪；黄龙滩电站扩能和松树岭、鄂坪、孙家滩电站建设进展顺利，潘口、龙背湾、孤山等电站的前期工作正抓紧进行；竹溪、竹山、房县、丹江口防洪工程建设顺利推进；东风金狮轮胎有限公司30万套全钢子午胎、丹江铝业公司电解铝、东风商用车公司精工齿轮等重点技改项目，以及秦岭山区农业综合开发、退耕还林、农村能源建设等项目正有序展开。

　　5、社会保持稳定。就业和社会保障工作取得新成效。认真落实再就业政策，广辟就业渠道，新增就业岗位2.3万个，安置下岗失业人员再就业2.19万人，城镇登记失业率控制在4.2%以内；积极筹措资金，确保了国有企业下岗职工基本生活费、企事业离退休职工养老金和符合享受失业保险待遇的失业人员救济金按时足额发放；城市居民最低生活保障制度进一步完善，低保政策全面落实。非典防治取得阶段性胜利。面对突发的非典疫情，全市上下全力以赴抗击非典，实现了创建“无非典安全区”目标。抗灾救灾工作主动扎实。全市周密部署，群策群力，战胜了汉江20年来最大洪水和局部暴雨、山洪等自然灾害，灾区群众生产生活得到妥善安置。社会治安秩序良好。强化综合治理责任制，深入开展“严打”整治斗争，依法打击“法轮功”等邪教组织，确保了全市治安秩序持续平稳。安全生产形势逐步好转。加强安全生产监管，建立重特大事故防范预警机制和基层安全生产监管体系，开展专项整治，重大安全事故得到有效遏制。

　　过去的一年，我市各项社会事业健康发展，政治文明和精神文明建设取得可喜成绩。全市落实“科技三费”1443万元，获得省年度科技进步奖11项，申报专利284项。基础教育、职业教育和成人教育协调发展，高考上省线人数突破万人大关。启动文化体制改革，加大文物保护力度，文化产业迅速发展。加强县乡村光纤网络建设，广播电视无线混合覆盖率达92%，有线电视用户增长17.4%。开展全民健身活动，发展竞技体育，在第十一届省运会上取得优异成绩。加强公共卫生体系建设，城区区域医疗中心地位进一步提升，农村卫生事业健康发展。计划生育实现省定目标，人口自然增长率为6‰。开展“做文明市民，树十堰形象”主题教育活动，精神文明创建更加贴近经济建设，贴近人民群众生活。科技、文化、卫生“三下乡”活动取得成效。强化全民国防教育，深入开展“双拥”活动，积极支持部队和国防建设，拥军优抚安置政策全面落实，实现了国家和省“双拥模范城”四连冠目标。坚持定期向人大报告工作，向政协通报情况，重大决策征求民主党派、党外人士、人民团体和咨询研究部门意见，推进政府决策的民主化、科学化。认真办理人大代表建议和政协委员提案，办结率达到100%。加强信访工作，密切了政府与人民群众的联系。深入开展“四五”普法活动，推行部门执法责任制和执法过错责任追究制，执法水平普遍提高。认真组织并完成了县市区及第五次村委会换届选举工作，基层民主政治建设得到加强。坚持开展民主评议行风活动，加大领导干部任期经济责任审计和大案要案查处力度，源头治腐工作向纵深推进。

　　在全市物质文明、政治文明和精神文明建设中，人事、外事侨务、民族宗教、统计、机关事务管理、档案、邮政、电信、地震、气象、水文、对台、社科、老龄、残联、地方志等方面的工作都取得了新的成绩。

　　各位代表，2003年是不平凡的一年。这些成绩的取得，凝聚了全市人民的智慧和力量！在此，我代表市政府，向辛勤工作在全市各条战线上的广大劳动者，向给予政府工作大力支持的人大代表、政协委员和各民主党派、工商联、无党派人士，向为我市发展作出突出贡献的东风公司、东风实业公司、东风金狮轮胎公司、汉江集团等大型企业，向驻市解放军、武警部队，向所有关心支持我市建设的海内外朋友，表示崇高的敬意和衷心的感谢！

[FS:PAGE]

　　在总结成绩的同时，我们也清醒地认识到，我市经济社会发展仍存在不少突出问题：经济的体制性和结构性矛盾依然突出，中小企业发展不快，民营经济规模小，经济外向度不高，整体竞争力不强；农业基础比较脆弱，非农产业发展不够，农产品加工滞后，农业产业化程度低，农民收入增长缓慢；固定资产投入规模小，重大项目、生产性项目、高新技术项目不多，经济增长后劲不足；就业再就业压力很大，社会保障体系不够健全，部分下岗失业人员、城镇低收入居民和贫困地区群众的生活还比较困难；政府职能转变缓慢，为市场主体服务的效率不高，少数政府工作人员还存在着官僚主义、形式主义、作风粗暴和腐败行为等等。对此，我们将采取有效措施，努力加以解决。

　　二、2004年工作的总体思路和主要措施

　　今年是实现“十五”计划的关键年，也是全面落实党的十六届三中全会精神的第一年。做好今年的各项工作具有十分重要的意义。我市发展既面临诸多有利条件，也存在一些不利因素。有利条件：一是我国正处于经济周期的上升阶段，国家继续实施积极的财政政策、新阶段扶贫开发政策，国债和新增财政资金重点向老工业基地倾斜、向农业农村倾斜，将对我市经济社会发展产生积极影响；二是发达国家和先进地区的资本在我国内地选择投资项目，有利于我市招商引资；三是东风公司改造十堰基地，实施中期事业计划，着力打造东风商用车国际品牌，有利于主导产业的发展壮大；四是南水北调中线工程启动，汉十高速公路明年上半年全线贯通，十漫高速公路开工建设，将对我市经济社会发展产生较强拉动作用；五是电力市场供求矛盾加剧，有利于汉江堵河流域水电开发。不利因素譬如：十堰经济支撑点比较单一，商用车的产品结构还没有完全调整到位，汽配企业调整步伐不快，一时还难以适应汽车产品结构调整的要求；黄姜产业面临着市场风险；近两年投资新上的项目还没有完全形成生产能力。综合考虑上述因素，制定积极而又稳妥的计划，既可以保证实实在在的速度，又可以争取效益与速度同步、当前与长远协调，实现可持续发展。

　　根据当前形势，今年我市经济社会发展的总体要求是：以邓小平理论和“三个代表”重要思想为指导，认真贯彻落实党的十六大和十六届三中全会精神，坚持以大开放总揽经济工作全局，以提高经济增长速度和效益、增加城乡居民收入、增强发展后劲为目标，着力改善发展环境，推进体制创新和科技创新，加快经济结构战略性调整，大力支持企业合资重组，加大招商引资和重大项目实施力度，切实做好服务东风公司和南水北调工作，促进国民经济持续快速健康发展；正确处理改革发展稳定的关系，推进社会进步，加快全面建设小康社会的步伐。

　　主要预期目标是：本市生产总值增长7%左右；全社会固定资产投资增长15%；社会消费品零售总额增长10%；财政收入增长8%，其中地方一般预算收入增长4%以上；城镇居民人均可支配收入增长7%，农民人均纯收入增长5%；外贸出口增长5%，利用外资增长20%；人口自然增长率控制在6‰以内；城镇新增就业岗位2.3万个，介绍安置就业2.5万人，城镇登记失业率控制在4.5%以内。

　　根据上述指导思想和奋斗目标，今年重点抓好以下几个方面的工作。

　　(一)大力实施工业兴市战略，加快改革调整步伐。

　　发展壮大汽车主导产业。一是全力支持“新东风”实施中期事业计划。把服务“新东风”当作当前头等大事来抓，广泛开展订单服务，提高服务的针对性和时效性；进一步做好政企共建工作，认真落实各项优惠政策，支持东风公司剥离辅体和分流人员，为推进国际合作营造良好环境。二是研究制定汽车配套产业发展规划，科学指导汽配产业发展。三是推进市属企业改革重组。规范企业民营化改革的操作程序，将资产面向社会公开出售。引导完成改制的企业按照现代企业制度的要求规范运作。推进汽车零部件企业与国内外优势企业联合重组，支持通达集团与万向集团合资合作、马来西亚金狮集团独资经营东风轮胎公司；支持市属骨干企业通过兼并、收购等方式，实现生产要素的优化配置。四是支持双鸥集团、太平集团、三环专汽、华阳集团和湖北电器厂等省属企业的改制重组。五是支持白浪汽配城发展汽车配件和整车市场贸易，扩大销售总量，增强汽配城的市场竞争力和社会影响力。充分发挥国家火炬计划汽车关键零部件产业基地的作用，走产业集群之路，做大做强汽车产业。

　　大力发展新兴支柱产业。以汉江堵河流域综合开发为重点，加快建设一批大中型电站。推进黄姜加工企业联合重组，切实解决低水平重复建设和无序竞争问题；搞好黄姜的良种选育和深度开发，提高种植、加工的科技含量，延伸产业链条。做好中药材基地的规范种植，重点抓好丹参、板兰根、黄连等中药材的基地建设和医药加工企业、医药流通企业的达标认证工作。确保水电和生物医药产业增幅20%以上。利用丰富的特色资源，大力开发有机茶、食用菌、山野菜、畜禽等骨干品种，突破性发展农产品加工业；组织企业申报认证绿色食品、有机食品品牌，加强市场营销，全力扩大市场份额。同时，用高新技术改造提升冶金、建材、纺织、化工等传统产业。引导企业实施品牌战略，增强产品市场竞争力。

　　抓好高新技术开发区和工业园区建设。围绕产业特色和区域特色，搞好园区规划，完善配套设施。按照打造现代制造业密集区的要求，充分发挥十堰高新技术开发区和东城、西城工业园区的龙头作用。以招商引资为着力点，促进产业链相关企业向开发区和工业园区聚集，引导产业集群和集约化发展，形成各具特色的园区经济。

　　（二）坚持“一主三化”方针，加速县域经济发展。

　　大力发展民营经济。放宽市场准入，落实投融资、税收、土地使用和对外贸易等方面的优惠政策，进一步优化民营经济发展环境。鼓励支持民营企业购买、兼并、承包和租赁国有集体企业；鼓励民间资本进入经营性基础设施和公益性事业领域；支持民营企业参与农业产业化经营，加快构筑县域经济以民营经济为主体的发展格局。

　　大力推进农业产业化经营。按照“区域化布局、规模化发展、产业化经营”的原则，大力发展黄姜、魔芋、茶叶、柑桔、蔬菜、中药材、食用菌、草食畜牧业等特色产业，建设高质量、高标准特色产品基地，逐步形成集中连片的特色产业带;着力引进和培植龙头企业，增强辐射带动力;发展农民专业协会、专业合作社等各种类型的经合组织。深化农业科技推广体制改革，积极探索按照特色产业布局，一个县市设置若干个由县市管理的区域性特色作物技术推广站。加大技术推广力度和重点科技攻关力度，提高特色产业科技含量；加强市级农产品质量监测与控制中心建设，推进农产品标准化体系建设，提高特色农产品的市场竞争力。

　　大力推进农村工业化。积极引导县市有重点地培育发展几个基础较好、特色突出、具有发展潜力的产业和企业；推动县市企业与大企业大集团合资合作、配套协作，实现产业连锁和规模扩张；加快推进县市中小企业产权制度改革步伐，建立健全中小企业服务体系，发挥有限财政资金的引导作用，支持县市支柱产业和骨干企业发展。

　　大力推进农村城镇化。切实搞好小城镇建设规划，统筹城乡经济社会发展。坚持以产业为支撑，走城镇建设、市场建设、工业园区建设相结合的发展路子。坚持“以业兴镇，以镇促业”，引导和鼓励各类企业向城镇集中，促进农村剩余劳动力在城镇寻找就业机会。利用工程移民、生态移民和迁移扶贫，争取国家优惠政策，鼓励农民进镇务工经商。

　　保护和提高粮食综合生产能力。进一步优化支农资金结构，保留水利基础设施建设以奖代补资金和温饱工程补贴款。增强粮食安全意识，实行最严格的耕地保护制度，加强基本农田保护。稳定粮食播种面积，依靠科技，提高单产，增加总产。

[FS:PAGE]

　　做好新阶段扶贫开发工作。适当调整扶贫重点村布局，新选63个边远贫困村作为今年建设对象，保持首批283个重点村建设规模。大力推进产业化扶贫，实施迁移扶贫，落实科技扶贫，把扶贫开发与农业结构调整、农村基础设施建设结合起来。加强扶贫资金管理，提高扶贫资金的使用效益，对扶贫资金实行公示、公告、报账制度。继续抓好农村饮水工程建设。健全和完善以农民工就业信息服务网络、就业培训系统、维权服务系统为重点的农民工跨地区就业服务体系，为农民外出务工提供服务，促进打工经济持续健康发展。

　　全面落实农村政策。认真贯彻落实中央一号文件精神，采取切实措施增加农民收入。稳定和完善家庭承包经营制度，加强对农村土地承包和流转的指导。放活山场水面经营权，加大山水资源开发力度。认真落实对农业和农户的信贷支持措施，增加农户小额信用贷款和农户联保贷款，大力发展农村经济。按照“减轻、规范、稳定”的要求，继续抓好农村税费改革，稳步推进乡镇综合配套改革，积极化解乡村债务。严格执行涉农收费价格公示制、农村义务教育一费制、村级公费订阅报刊限额制和农民负担责任追究制，确保农民负担不反弹。

　　（三）努力扩大投资、消费、出口，增强经济发展后劲。

　　努力扩大投资。一是全方位招商引资。抢抓当前国际资本和沿海产业资本向中西部转移的机遇，重点围绕制造业和旅游业，着力引进大企业、大集团。加大代理招商、网络招商和以商招商力度，开展形式多样的招商活动，力争取得实效。继续实行和完善招商引资目标责任制，积极构建政府引导、企业为主、中介推动、社会支持的新型招商引资运行机制。二是争取国家建设资金。根据国债发行的重点，有针对性地开展项目争取工作，从财力上保证项目的前期准备工作，确保每年都能储备、立项和开工一批项目。三是扩大民间投资。按照“非禁即入”的要求，进一步放开投资领域，激活民间资本，力争民间投资有较大幅度的增长。四是全力推进重点项目建设。确保黄龙滩电站扩能、孙家滩电站、开泰激素系列新药、普林工业园汽车配件、丹江电解铝、农夫山泉果汁饮料和东风大道等项目年内竣工。加快秦岭山区农业综合开发、农村能源、丹江二桥、县乡路网改造、松树岭电站、鄂坪电站、东风全钢子午胎等续建项目建设进度。力争十漫高速公路、东风实业汽车灯具、三环1.5万辆汽车制造、丹江钢铁公司30万吨型材、三里坪电站、将军河大桥等项目开工建设。

　　积极扩大消费。加快发展旅游业。充分利用襄十高速公路贯通、南水北调中线工程开工等有利条件，挖掘整合以武当山为龙头的旅游资源，开发生态、人文和环保旅游产品，大力宣传促销，确保全年旅游人数和收入增长20%以上。发展现代商贸流通业。鼓励大型商贸流通企业利用连锁经营、网上购物、网络服务等新型服务方式，积极开拓周边市场。积极引进国内外著名物流企业，培育建设物流园区，积极筹建鄂西北小商品市场，加快区域性物流中心建设步伐。培育和扩大住房、汽车、信息服务等新的居民消费热点。放开搞活住房二级市场，盘活城市存量房产，促进住房消费；清理车辆使用中的各种收费，简化购车和年检手续，加快城市道路、停车场等配套设施建设，促进个人汽车消费；建立健全文化、体育、医疗保健、物业管理等社区服务网络，推动消费结构升级。

　　进一步扩大出口。加快十堰海关建设，力争年内开关。建设特色产品出口基地，引进和培养外经贸专业人才，帮助企业申办进出口经营权，落实鼓励外贸出口的各项优惠政策，鼓励企业积极开拓国际市场，扩大出口份额。

　　（四）推进财税改革，做好财税金融工作。

　　加强财税工作。加快“金财”、“金税”工程建设，对重点行业、重点税源、重点税种实施有效监控，完善征管体系，加大征管力度，实现应收尽收。调整和优化支出结构，重点向解决“三农”问题、扩大就业、完善社会保障体系等方面倾斜，确保机关、事业单位职工工资和离退休人员离退休费按时足额发放，保证机关正常运转。完善公共财政体制，继续推进部门预算、国库集中收付、政府采购和收支两条线管理改革。进一步加强审计监督。

　　完善金融服务。加强同金融部门的沟通与衔接，配合处理不良债务，积极推荐优质项目，扩大利用银行信贷投资。以创建“信用县市”和“信用企业”为基础，大力推进“信用十堰”建设。建立以中小信用担保机构为主体、商业性担保机构为补充的担保体系，以解决中小企业和民营经济担保难、贷款难问题。稳步推进农村信用社改革，管好用好支农再贷款。

　　（五）做好就业再就业工作，健全社会保障体系。

　　千方百计扩大就业。牢固树立就业是民生之本的思想，全面落实各项促进就业的优惠政策，引导下岗失业人员和剩余劳动力到劳动密集型产业、社区服务业和民营企业就业，拓宽就业渠道。加强劳动就业培训，注重劳动就业基地、劳动力市场和劳动保障信息网络建设，大力开发公益性就业岗位，优先安置有就业愿望的特困人员，特别是“4050”人员和双下岗失业人员再就业。

　　完善社会保障体系。继续做好“两个确保”和城市“低保”工作，严格将社会保障资金纳入财政预算，增加社会保障支出。规范社会保障资金管理，确保国有企业下岗职工基本生活费和离退休人员基本养老金按时足额发放；确保失业人员按规定足额享受失业保障金待遇；确保符合条件的城镇贫困人口全部纳入最低生活保障范围。进一步扩大养老、失业、医疗、工伤、生育保险覆盖面。加强劳动用工监察，严肃查处拖欠务工人员工资的行为，维护务工人员合法权益。

　　建立社会帮扶和救助体系。建立救灾工作分级负责制、救灾经费分级负担制和社会动员机制。推进农村五保“福星工程”，逐步实现乡镇集中供养。高度重视人口老龄化问题，维护老年人的合法权益，逐步提高老龄事业的投入。关心群众生活特别是困难群众的生活，做好特困群众的救助工作。

　　（六）坚持生态立市,加强园林城市建设和管理。

　　加强生态环境建设。严格执行退耕还林政策，巩固退耕还林和封山育林成果，做好天然林保护工作，积极争取丹江口库区湿地保护项目。争取农村沼气国债建设项目，完成“一建三改”沼气池1.5万口。继续抓好小流域综合治理，治理水土流失面积280平方公里。严格实行环境影响评价、环保“三同时”、排污申报和许可证制度，加强重点污染源治理，抓好城市环境功能区达标。认真研究制订黄姜加工新工艺，尽快达到污水排放和“三废”综合利用标准。加强地质灾害防治，建立地质灾害防控体系。

　　加大城市建设管理力度。按照区域性大城市的要求，加快十堰大城市的建设步伐。科学规划城市功能布局，优化城市路网结构，扩大城市发展空间，全面提升城市功能。加快东风大道、天津路、重庆路、大岭路、甘肃路的建设步伐。建立多元化的投资、建设、运营模式，抓好城市供水、供热、供气和污水处理等基础设施建设。全面推行绿化养护和环卫保洁综合承包责任制，实施绿化、净化、美化、亮化工程，提高园林绿化品位。争创国家生态城市和卫生城市。

　　服务南水北调中线工程。继续做好水污染防治和水土保持工作；配合长江委编制好移民规划；扎实做好工程补偿项目前期工作，争取纳入国家相关规划；做好大坝施工区的服务工作；妥善处理老库区移民遗留问题。

　　（七）加强精神文明建设，推进社会事业发展。

　　深入开展精神文明创建活动。抓好文明单位（行业）创建活动，将文明创建与“内强素质、外树形象”结合起来；抓好文明村镇创建活动，实施“扶贫、信用、文化、生态、健康、平安”工程，提高农民生活质量；抓好文明城市创建活动，活跃社区文化，美化人居环境，提高城市文明程度。深入开展国防教育，加强对民兵预备役工作的领导，积极支持部队和国防建设，进一步巩固全国、全省“双拥模范城”成果。

[FS:PAGE]

　　全面加强人才工作。牢固树立“人才资源是第一资源”的观念，加强人才资源能力建设，发挥人才资源开发在经济社会发展中的基础性、战略性、决定性作用。大力培养各级各类人才，建立公开、平等、竞争、择优的选拔任用机制，促进优秀人才脱颖而出。开辟人才引进和流动的“绿色通道”，重点吸引经济社会发展急需的高素质人才和专业人才。完善鼓励人才创新创造的分配制度和激励机制，落实知识、技术、管理等要素参与分配的政策，重奖有突出贡献的各类人才。抓紧制定人才工作规划，完善政策，营造环境，努力做到用事业造就人才、环境凝聚人才、机制激励人才、法制保障人才。充分利用东风公司等中央、省属大型企业的人才资源优势，为加速十堰经济社会发展服务。

　　大力实施科教兴市战略。组织实施水源区可持续发展、汽车关键零部件、生物医药和制造业信息化等科技专项行动计划，加快科技成果向现实生产力转化；支持企业与高等院校、科研院所合作，开发具有自主知识产权的核心技术，力争专利申请突破300项；探索建立以政府投入为引导、企业投入为主体、社会资本和风险投资为补充的科技投入体系。积极推进教育改革与发展，全面实施素质教育。将教育工作的重点放在农村，放在义务教育和职业教育上；巩固和完善地方政府负责、分级管理、以县为主的管理体制，增加对教育特别是贫困地区义务教育的投入；实施“三教统筹”，大力发展农村初二后分流教育和多种形式的职业技术教育；加强教师队伍建设，改善办学条件，提高办学质量和水平；适应城镇化、初中渡峰和逐步普及高中阶段教育的需要，整合教育资源，优化学校布局；抓好学校危房改造和食品卫生工作，高度关注学生安全。继续推进综合性大学建设。

　　发展文化体育卫生事业。加快文化体制改革步伐，推进非公益性国有文化资产向经营性文化资产转化；组建广电和文化出版发行实体，重组专业文化经营公司；搞好基层公共文化设施建设，加强音像制品和书报刊管理，整顿网络市场和文化娱乐市场秩序。依法加强文物保护工作，积极筹建市博物馆。大力开展全民健身运动，不断提高全民身体素质和竞技体育水平，发展体育产业。加强公共卫生设施建设，完善公共卫生应急、疾病预防控制和卫生监督执法等体系；改善农村医疗卫生条件，办好新型农村合作医疗制度改革试点。

　　切实抓好人口与计划生育工作。加强县乡村三级计划生育技术服务网络建设。加强舆论引导，改变重男轻女的落后观念；加大执法力度，严厉打击利用非法手段为他人进行胎儿性别鉴定和选择性别终止妊娠行为。建立和完善以现居住地管理为主的流动人口计划生育管理和服务工作机制，做好流动人口计划生育管理工作。

　　（八）加强政治文明建设，提高政府依法管理经济和社会事务的水平。

　　大力推进民主法制建设。严格按照法定的权限和程序履行职责，认真执行人大的各项决议，自觉接受人大监督，接受政协民主监督；认真听取各民主党派、工商联、无党派人士、人民团体和非公有制代表人士的意见；建立和完善重大问题集体决策制度、专家咨询制度、社会公示和社会听证制度，提高决策的科学化、民主化水平；依法健全村民委员会的直接选举制度、村民议事制度、村务公开制度。继续开展“四五”普法教育，增强全民的遵纪守法、依法维权意识。认真贯彻实施《行政许可法》，做好《行政许可法》的宣传培训工作，全面清理和规范行政许可文件、行政许可项目和行政许可主体。严格执行执法责任制和执法违法责任追究制，促进公正执法、严格执法和文明执法。

　　切实做好社会稳定工作。认真做好群众来信来访和人民调解工作，按照“分级负责、属地管理”的原则，努力把各种矛盾化解在萌芽状态，积极预防和妥善处置突发性和群体性事件。加强社会治安综合治理，构建治安防范体系，依法严厉打击各种违法犯罪活动，确保人民群众安居乐业。高度重视和切实做好安全生产工作，防止重特大事故发生。采取有效措施，做好高致病性禽流感的防控工作。

　　积极转变政府职能。依法履行政府职责，转变政府调控和监管方式，主要运用经济和法律手段管理经济社会事务，切实把政府职能转到经济调节、市场监管、社会管理和公共服务上来。继续开展行风评议和企业评议政府部门活动，推行政务公开，提高办事效率，优化投资环境，努力建设服务型政府。切实改进工作作风，密切联系群众；坚持实现好、维护好最广大人民群众的利益，凡是得民心、顺民意的事都竭尽全力去做，凡是失民心、违民意的事都雷厉风行去改，真正做到权为民所用，情为民所系，利为民所谋，努力建设民生政府。坚持克服和纠正权责分离、权责不清和只要权力不要责任的现象，树立设权应法定、有权必有责、用权受监督、违法要追究、侵权需赔偿的观念，强化政府工作人员的责任意识，努力建设责任政府。发扬求真务实的精神，坚持讲实话、察实情、办实事、求实效，反对虚报浮夸，大力精减会议、文件，减少事务性应酬，把更多的时间、精力放在帮助解决基层和群众的实际问题上，切实解决好人民群众关心的实际问题，努力建设务实政府。

　　坚定不移地加强廉政建设。按照“为民、务实、清廉”的要求，坚持标本兼治、惩防并举，全面推进反腐倡廉工作。继续落实领导干部廉洁自律的各项规定，进一步规范从政行为；继续加大违纪违法案件查办力度，坚决惩处腐败分子；继续做好纠风专项治理工作，切实加强政风建设；继续推进体制创新和制度建设，从源头上预防和治理腐败。加大治理教育乱收费力度，加大纠正医药购销和医疗服务中的不正之风力度，加大减轻农民负担工作力度，加大安全生产管理和事故责任追究力度，把纠正和查处严重损害群众利益的不正之风作为廉政建设的重点工作，切实抓出成效。

　　各位代表，做好2004年的工作，对于我市顺利实现“十五”计划，全面建设小康社会，意义十分重大。2004年也是本届政府任期的最后一年，我们有信心、有决心，在市委的正确领导下，振奋精神，团结拼搏，克服困难，开拓前进，努力完成全年各项目标任务，向全市人民交上一份满意的答卷！
